# Sensing of Microvascular Vasomotion Using Consumer Camera

**DOI:** 10.3390/s21186256

**Published:** 2021-09-18

**Authors:** Itaru Kaneko, Yutaka Yoshida, Emi Yuda, Junichiro Hayano

**Affiliations:** 1 Center for Data-Driven Science and Artificial Intelligence, Tohoku University, Sendai 980-8576, Japan; emi.a.yuda@tohoku.ac.jp; 2 Graduate School of Design and Architecture, Nagoya City University, Nagoya 467-8601, Japan; yoshida@sda.nagoya-cu.ac.jp; 3 Heartbeat Science Lab Co., Ltd., 6-6-40 Aoba, Aramaki Aoba-ku, Sendai 980-8579, Japan; hayano@acm.org

**Keywords:** vascular, blood, MVW, vasomotion

## Abstract

In this paper, we will introduce a method for observing microvascular waves (MVW) by extracting different images from the available images in the video taken with consumer cameras. Microvascular vasomotion is a dynamic phenomenon that can fluctuate over time for a variety of reasons and its sensing is used for variety of purposes. The special device, a side stream dark field camera (SDF camera) was developed in 2015 for the medical purpose to observe blood flow from above the epidermis. However, without using SDF cameras, smart signal processing can be combined with a consumer camera to analyze the global motion of microvascular vasomotion. MVW is a propagation pattern of microvascular vasomotions which reflects biological properties of vascular network. In addition, even without SDF cameras, MVW can be analyzed as a spatial and temporal pattern of microvascular vasomotion using a combination of advanced signal processing with consumer cameras. In this paper, we will demonstrate that such vascular movements and MVW can be observed using a consumer cameras. We also show a classification using it.

## 1. Introduction

In this paper, we will introduce a method for detecting microvascular waves (MVW) extracted from the image in the video taken with consumer cameras. Fluctuations in micro vessels are a dynamic phenomenon and can fluctuate over time at frequency below 1 Hz for variety of reasons [1]. Further, its sensing is used for variety of purposes [2,3]. For those purposes, the special device, side stream dark field cameras (SDF cameras) were developed in 2015 for medical purposes [4].

On the other hand, Sugita et al. measured blood vessels from video images. Studies by Sugita et al. have shown that it is possible to measure heart rate and blood pressure by photographing the skin with high frame rate cameras at high frame rates and analyzing color [5].

Therefore, it is possible to use this technique and use high frame rate cameras instead of SDF cameras. In other words, in this study, while using regular video cameras, Sugita et al. detected blood pressure and heartbeats, and this research enables a new application to find the spatial frequency distribution of the movement of capillaries. In addition, for the capturing of the autonomous movement of capillaries, we will provide new techniques to use normal cameras without directly inputting detailed images of capillaries using cameras. However, in our application, a high frame rate is not necessary.

The mechanisms to control such fluctuations are also studied in various aspects [6,7,8] and there are proposals to detect such fluctuations, e.g., one using the laser Doppler effect [9]. 

Our group is proposing the analysis of MVW as one of dynamic features of microvascular events and developing an analysis method for it [10]. As mentioned earlier, blood flow in microvessels fluctuate over time, however, these fluctuations are also organized as spatial and temporal propagations of waveform. MVW is a propagation pattern of microvascular vasomotions and statistical image analysis helps the analysis of vasomotion. Therefore, we aimed to implement the analysis method of video taken from above the skin to analyze MVW.

In our method, MVW is analyzed through several steps of the image processing. The diff images of captured video frames are calculated to enhance small color changes due to blood flow.

**Figure 1:**
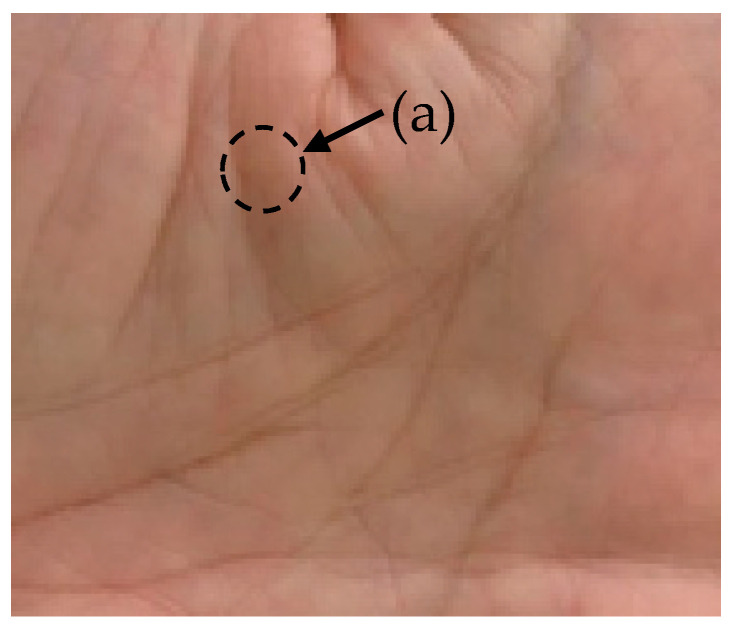
Single frame image of captured video. The dashed circle (a) shows an example of a mottled red pattern also found in other areas of the skin.

On the skin of hands, mottled red colored patterns are observed on all areas of skin and they change over time. Those are caused by the motion of capillary walls. Such a fluctuation of mottled red patterns has a temporal and spatial structure similar to small waves. This is not clear as you can see in the circle (a) in Figure 1. This is because it is a still picture. However, in the video, you can see it as the motion of mottled red color pattern on the skin. When they are observed, temporal changes of those mottled patterns are flickering with period between several ten seconds and one minutes. The observation of such spatial and temporal waveform of the blood vessel through the skin by regular cameras had not been evaluated since now.

We will show two experimental results: (1) Image processing of video to extract features which corresponds to the MVW. (2) Simple feature extraction and discrimination using that feature extraction. The discrimination experiment is planned to integrate with image processing of video though it is not yet fully integrated.

Our overall goal is to confirm the possibility of such imaging and measurement of MVW.

## 2. Subjects and Data Acquisition Methods of the Experiment

Two groups of subjects participated in the experiment. Group 1 consists of three young subjects. The ages were 32 ± 7 years old and consisted of one female and two males. Group 2 is a group of elderly subjects. They were 75 ± 7 years old and all male. Those people agreed to be subjects after being informed of the objective of the experiment. The set of subjects was limited because the experiment was still in the preparatory stage of the experiment. Further, our current goal is to detect MVW as a visual feature. The number of subjects was sufficient for that purpose.

Videos were recorded on the centers of the hands of subjects from a distance of 20 cm, using a video camera (Everio R, JVCKENWOD co., Ltd., Kanagawa, Japan). Figure 1 shows a frame in the recorded video.

The experiment was carried out in the quiet room with room temperature of 24 °C while hands were fixed on the table. Square areas of 3 cm and 5 cm were captured at a length of 9 minutes each. The videos recorded by CE cameras were usually formatted as MP4 format (standard file format contain AVC/H264 video) which is widely used among such products.

## 3. Feature Extraction through Image Processing

We implemented feature extraction using image processing using FFT (fast Fourier transform) applied to the video records. The purpose of this process was to extract video of vasomotion from captured video using FFT. We extracted all video frames and calculated FFT of entire video. We then applied several processing steps to calculate precise differential images.

Since hands move slightly at all time during the recording, a simple differential image may contain large noise energy caused by such a small movement. Therefore, motion compensation is required to reduce such noise. To compensate for such small movements, it is expected that correlation and transfer function of entire image will produce the best result.

**Figure 2:**
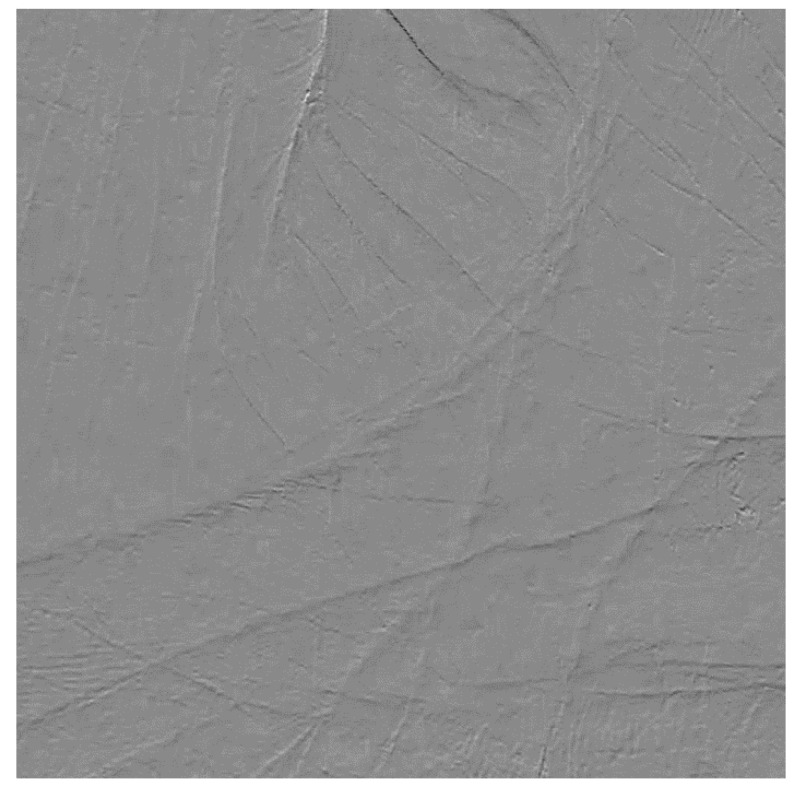
Differential image calculated by pixel accuracy alignment using cross correlation which contains some noise because of sub pixel error.

At first, we calculated a 2D (two dimensional) correlation of the entire image to find rough alignment. It derived a pixel accuracy alignment of the image. Figure 2 shows differential images calculated by pixel accuracy alignment using cross correlation.

That correlation contains multiple blurred peaks, instead of single sharp peak. We selected the maximum point as initial alignment. However, using that point as alignment, will leave subpixel misalignment error and cause additional noise corresponding to the subpixel misalignment, as shown in Figure 2. The original image has dark or glowing shadows along with lines in the original picture. Those are not difference in two frames but caused by the subpixel misaligned of two frames.

Subpixel motion compensation is required to eliminate subpixel errors. The 2D transfer functions, were calculated from the entire image and can be used for such subpixel motion compensation. However, the entire component of transfer function may contain meaningless components which correspond to the false similarity of other parts of the two images. To eliminate such a noisy element, it is very effective to apply a mask to the entire transfer function to pull out meaningful areas.

**Figure 3:**
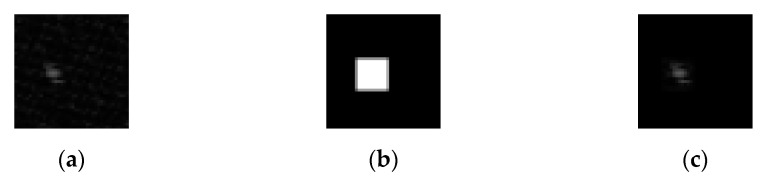
Calculation of transfer function for performing subpixel motion compensation. (**a**) The 2D transfer function of reference frame to target frame that contains noise due to false similarity. (**b**) The 9 × 9 2D rectangular window to pull out meaningful components of transfer function. (**c**) The filtered component of transfer function masked by the 9 × 9 mask.

Figure 3 shows the calculation of the transfer function for performing subpixel motion compensation. (a) The 2D transfer function of reference frame to a target frame that contains noise due to false similarity. The noise appears as dark mottled patterns spread across all area of the image. (b) The 9 × 9 2D square window was used to pull out meaningful components of transfer function, shown as white rectangular images in the figure. (c) Filtered component of transfer function masked by a 9 × 9 mask. By applying the mask (b) on the 2D image (a), the noise component is eliminated and this transfer function will give fine alignment of image of two frames.

**Figure 4:**
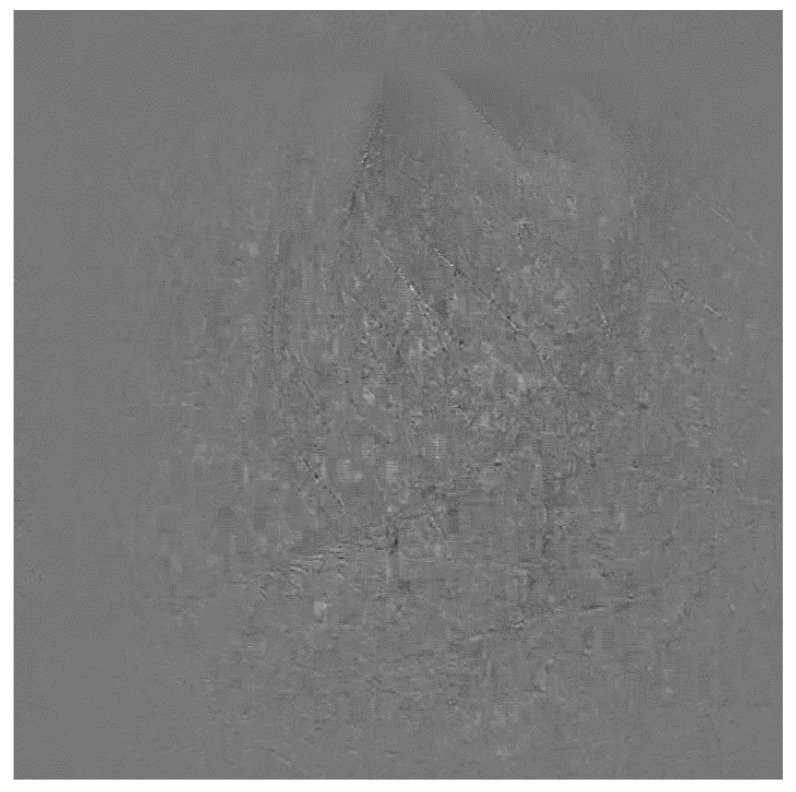
Differential image calculated by subpixel accuracy alignment using cross correlation.

By applying the transfer function, as a side benefit, average illumination and amplitude will be compensated within a single processing step of image processing, at the same time as motion compensation. The resulting image is shown in Figure 4. White or black shadows of edges were removed and only the difference of two frames remained. After sub-pixel motion compensation, a clear, noiseless differential image was derived. The edge noises, caused by misalignment, were effectively removed.

**Figure 5:**
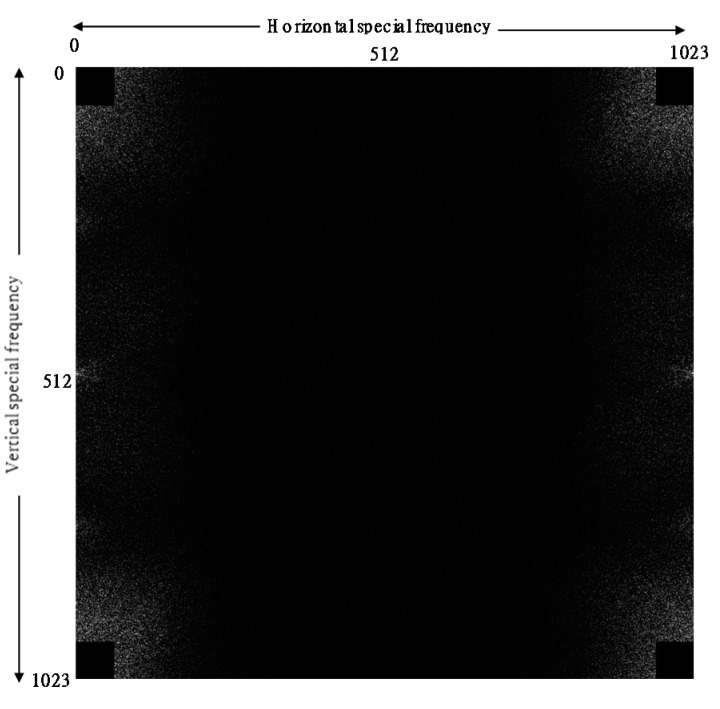
Two-dimensional auto correlation in frequency domain.

**Figure 6:**
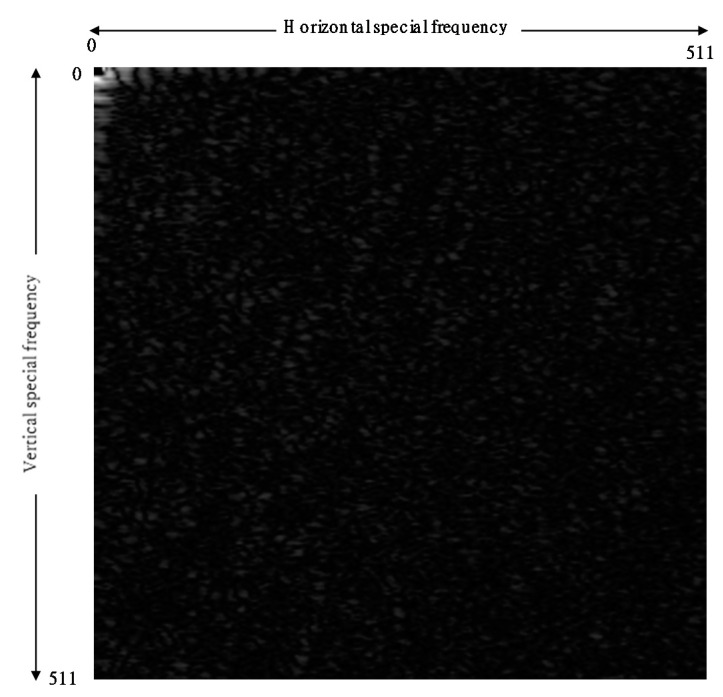
Two-dimensional auto correlation in spatial domain.

The spatial frequency spectrum of MVW was then calculated. Figure 5 shows calculated, two-dimensional auto correlation in frequency domain. There are 1024 × 1024 spatial frequency components on two axes. Due to the nature of frequency spectrum, four quadrants of the frequency spectrums are symmetrical. Therefore, we will show only first quadrant after this figure. We removed DCs (direct currents) up to 64 frequency lines. Therefore, four corners of spectrum are black. These low frequency bands reflect the shape of hands but do not correspond to the correlation of mottled pattern in nearby images. Figure 6 shows the result of two-dimensional, spatial domain auto correlation of the images. Since the four quadrants are symmetrical, the space is shown. In case of ‘Elderly1’, there is a bright pattern in upper left corner of the 2D spectrum. This is interpreted as it has peak around 4 Hz.

**Figure 7:**
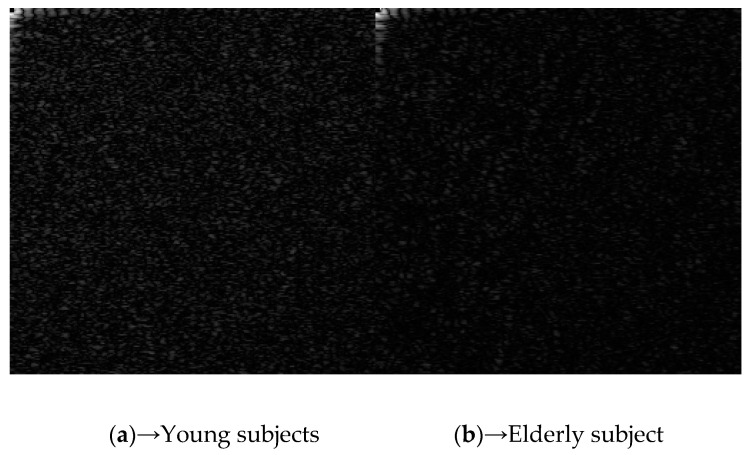
Comparison of (**a**) young (left) and (**b**) elderly (right) subjects.

Figure 7 shows the autocorrelations for MVW of (a) young (left) and (b) elderly (right) subjects. The resulting differences in the features of the 2D correlation pattern may include spatial and temporal characteristics corresponding to the blood vessels through the skin. We are not yet be able to analyze these features among different age groups. However, a clear frequency spectrum of mottled pattern has been derived.

## 4. Spatial and Temporal Feature Analysis

This chapter provides a simpler spatial and temporal analysis of the images to show the possibility to use those mottled pattern for the biological sensing. 

Features in the resulting 2D correlation pattern include spatial and temporal characteristics of the color that pass through the skin corresponding to the blood vessels. Furthermore, and from there, we can analyze biological properties. Observing the time and frequency patterns of the image using 2D correlation is useful for detailed analysis. However, it can also be performed with simpler spatial and time feature analysis.

In this simpler method, we extracted still image in every one second throughout the entire video.

We examined average RGB (red–green–blue) values for the circular area within radius R (red color) at the center of hands for each second. The average value for each second is handled as time series and power spectrum of the temporal transition of value was calculated. To compare different sizes of the area, we compared the r (radius) = 0,1,2,3,4,5,10,15 mm. For r = 0, we do not actually use area with r = 0 (which is impossible) and pick the red color of single pixel. We calculated low frequency band powers (0–0.25 Hz) and high frequency band powers (0.25–0.5 Hz) for each different radius from power spectrum. Further, the number of peak frequencies which exceeded average power were calculated for each radius. The calculated number became a feature of spectrum which relate to roughness etc. The analysis is compared for each color (R, G, B) because blood vessel visibility is different for each color.

**Figure 8:**
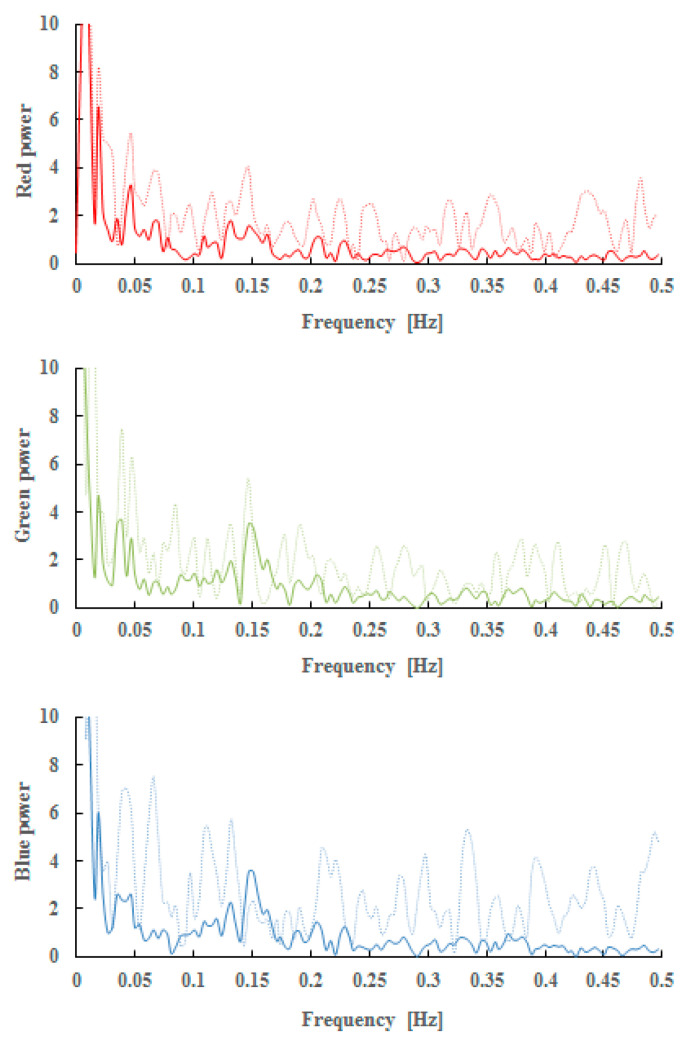
Power spectrum calculated from time series of the RGB average values (solid line: r = 0, dot line: r = 15).

Figure 8 shows the difference in power at each R value for r = 0 (corresponding to one pixel). Solid lines represent a low frequency band and thin dotted lines represent a high frequency band.

**Figure 9:**
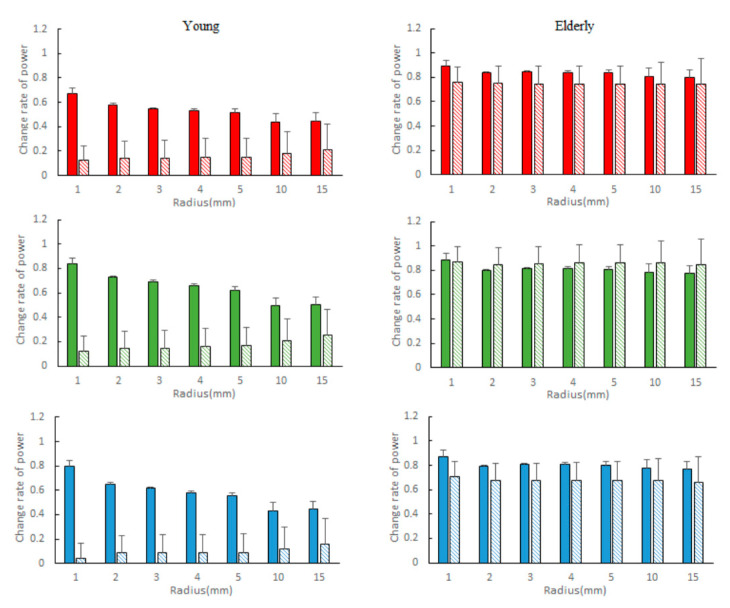
Difference in power at each R value for r = 0. Left: young (*n* = 3), right: elderly (*n* = 8) solid: low frequency band, fine dotted: high frequency band upper red power, middle green power, lower blue power, mean ± S.E.

Figure 9 compares the changes in radius r and power in the high and low frequency bands between the two groups. In the group of young subjects, the low frequency power decreased in power due to the increase in radius which was large. Especially in the high frequency range, the decrease was large. In young subjects, as the radius r increased, the low frequency power decreased moderately, and the power of the high frequency band increased. In contrast, in regard to the elderly subjects, as the radius r increased, power did not change clearly.

**Figure 10:**
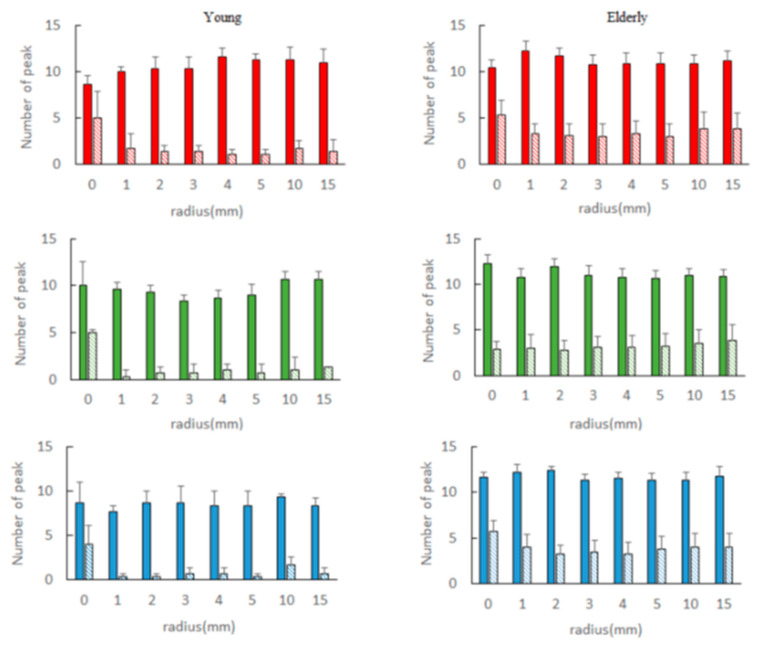
Number of different peak frequency for each radius. Left: young (*n* = 3), right: elderly (*n* = 8) solid: low frequency band, fine dotted: high frequency band, upper red power, middle green power, lower blue power, mean ± S.E.

Figure 10 compares the number of peaks when the frequencies are divided into smaller pieces. Younger subjects, as the radius r for red color increased, the number of peaks increased. In the case of the green color, it decreased to R = 3 and then increased again from R = 4. For the blue color, the number was less than that of red and green. On the other hand, the number of peak frequencies in the elderly group increased more than that of the young.

## 5. Discussion

We aimed to demonstrate method of observing microvascular wave (MVW) by extracting differential image from the video captured by consumer cameras. We also tried to show correlation between age groups and a features of MVW.

This study added new function of the spatial frequency distribution of the movement of capillaries to the analysis of video images from regular video cameras. Further, regarding the capturing of the autonomous movement of capillaries, we provided a new technique to use normal cameras without directly inputting detailed images of capillaries using SDF cameras.

In the feature extraction through image processing, as shown in Section 3, experimental result to process image using FFT to derive differential image of the frames are shown. Using FFT for the image processing to derive differential figure, the experiment has shown the sufficient performance of transform function and effectiveness of sub pixel alignment. Further, it was also confirmed that consumer cameras have performance to capture the MVW. It was also shown that noise caused by misalignment was effectively removed by the proposed method, by applying a rectangular mask in the frequency domain. 

Compared to the existing device, the frequency spectrum of MVW was measured without specialized lighting, and specialized a jig to fix the camera against hands or a faster frame rate to observe MVW. However, we are not yet confirmed that extracted feature is adequate for the various classification or analysis. We need further experimented with larger number of stimulus. This experiment only demonstrated the ability to extract the frequency domain feature of MVW.

In Section 4, we presented experimental results of spatial and temporal feature analysis. We have shown that the difference of elderly subjects and young subjects can be identified by spatial and temporal feature analysis. Since integration of feature extraction is not fully integrated, we still need work to integrate the discrimination method with feature extraction to fully gain from the advantage of our feature extraction method.

The experimental results to find the correlation with age and MVW (Section 4) has the following restrictions. Firstly, there is a problem of samples and selection. In this study, the data of the subjects were measured using the probability sampling method, but it cannot be denied that sampling errors occur. Therefore, it does not necessarily reflect the general population or the appropriate population. The subjects of the study are limited to Asian races due to their geographical area, and as such, there is a selection bias. Secondly, the sump size of statistical measurements is inadequate. It is necessary to use a sufficient sample size to assemble valid research results, but in this study, the samples are small and it is somewhat difficult to identify important relationships from the data. Larger sample sizes are required for larger populations to be suitable.

Because the number of younger subjects was only three, it is insufficient to state any statistically reliable conclusion. Furthermore, the discrimination method is not yet fully integrated with the proposed image processing method.

It has already been shown in the previous research [3] that the movement of capillaries is useful for medical purposes. On the other hand, the movement of capillaries shown to be able to be analyzed in this study is not as clear as that obtained by SDF cameras, but only its spatial frequency distribution. In order to use it for medical measurement, it is necessary to investigate the correlation between the observed frequency distribution and the state of the living body. For example, investigating the correlation with age is the first step, but the future direction is to determine the correlation between the spatial frequency distribution of capillary movement, including this, and the biological state.

Our next step is to integrate image processing method with the MVW based discrimination method. It is also necessary to increase number of subject and achieve statistically sufficient result.

## 6. Conclusions

It was shown that consumer cameras can be used to capture microvascular vasomotion using image processing of video using FFT, which can effectively remove noise and error because of camera motion.

From those results obtained by temporal and spatial analysis, it is considered that with younger subjects, each pixel’s opening and closing of the skin blood vessels did not synchronize and had different frequencies. That is a possible reason for the decrease in power of a wider size of area. Especially true, for the low frequency band, more frequency lines will be expected to be observed when the size of the areas are widened. In contrast, it is mentioned that the opening and closing phase are synchronized in the elderly even when observing the wider size of the area.

The limitation of this research is still the small number of subjects which makes it difficult to derive a valid statistical result. Because we are currently focusing on the extraction of the image of MVW, we are not yet comparing statistical analyses between the MVW and clinical data using massive data. This is a future research goal.

We have shown the possibility that MVW can be detected and image-analyzed with a simple device. As a result, various features appearing in MVW may be used in medical applications in the future. In addition, although this research made it possible to detect MVW using a simple imaging device by image processing, it is possible that the same method can be applied to improve the performance of existing imaging devices. We plan to continue research on these issues.

## Data Availability

Anonymized data are available on request to the corresponding author.
